# Upregulation of the serine palmitoyltransferase subunit SPTLC2 by endoplasmic reticulum stress inhibits the hepatic insulin response

**DOI:** 10.1038/s12276-022-00766-4

**Published:** 2022-05-05

**Authors:** Goon-Tae Kim, Shivani Devi, Amitesh Sharma, Kyung-Hee Cho, Su-Jung Kim, Bo-Rahm Kim, Sang-Ho Kwon, Tae-Sik Park

**Affiliations:** 1 Department of Life Science, Gachon University, Sungnam, Korea; 2 Biomedical Research Center, Asan Institute for Life Sciences, Seoul, Korea; 3 The Institute of Vision Research, Department of Ophthalmology, Yonsei University College of Medicine, Seoul, Korea; 4 Department of Cellular Biology and Anatomy, Medical College of Georgia, Augusta University, Augusta, GA USA; 5 Lipidomia Inc., Sungnam, Korea

**Keywords:** Metabolic disorders, Metabolomics

## Abstract

Endoplasmic reticulum (ER) stress is induced by various conditions, such as inflammation and the presence of excess nutrients. Abnormal accumulation of unfolded proteins leads to the activation of a collective signaling cascade, termed the unfolded protein response (UPR). ER stress is reported to perturb hepatic insulin response metabolism while promoting insulin resistance. Here, we report that ER stress regulates the *de novo* biosynthesis of sphingolipids via the activation of serine palmitoyltransferase (SPT), a rate-limiting enzyme involved in the *de novo* biosynthesis of ceramides. We found that the expression levels of Sptlc1 and Sptlc2, the major SPT subunits, were upregulated and that the cellular concentrations of ceramide and dihydroceramide were elevated by acute ER stress inducers in primary hepatocytes and HepG2 cells. Sptlc2 was upregulated and ceramide levels were elevated by tunicamycin in the livers of C57BL/6J wild-type mice. Analysis of the Sptlc2 promoter demonstrated that the transcriptional activation of Sptlc2 was mediated by the spliced form of X-box binding protein 1 (sXBP1). Liver-specific Sptlc2 transgenic mice exhibited increased ceramide levels in the liver and elevated fasting glucose levels. The insulin response was reduced by the inhibition of the phosphorylation of insulin receptor β (IRβ). Collectively, these results demonstrate that ER stress induces activation of the *de novo* biosynthesis of ceramide and contributes to the progression of hepatic insulin resistance via the reduced phosphorylation of IRβ in hepatocytes.

## Diabetes: Linking a lipid to insulin resistance

A lipid molecule called ceramide is key to regulating the body’s insulin response, which controls blood sugar, and thus may hold keys to new treatments for metabolic diseases such as diabetes. Although ceramide levels were known to be raised in obesity and diabetes, the mechanism remained unclear. Tae-Sik Park at Gachon University, Sungnam, South Korea, and Sang-Ho Kwon at Augusta University, USA, and co-workers investigated how excess ceramide production is triggered and the blood sugar regulation consequences. They found that the liver-specific SPTLC2 transgenic mice fed a high-fat diet had increased levels of an enzyme activity of serine palmitoyltransferase which led to synthesis of high levels of ceramide in the liver. The high ceramide levels suppressed insulin signaling, imbalancing blood sugar levels and causing liver toxicity. Therapies that inhibit ceramide synthesis show promise for treatment of metabolic diseases.

## Introduction

Overnutrition-induced obesity is a risk factor for metabolic diseases, such as type II diabetes, hypertension, and coronary heart disease^1–3^. Elevated plasma free fatty acids (FFAs) lead to unregulated uptake by the liver, resulting in the development of nonalcoholic fatty liver disease^4^. Elevated levels of FFAs contribute to the induction of insulin resistance and diabetes by modulating glucose and lipid metabolism in skeletal muscle, liver, and adipose tissues^5^. Under obese conditions with excess FFAs in the circulation, saturated fatty acids (FAs) contribute to lipotoxicity via the synthesis of specific lipid metabolites that act as signaling molecules in response to saturated FFAs^6^. These lipid metabolites include diacylglycerol (DAG), ceramide or their metabolites that activate serine/threonine kinases or phosphatases that inhibit the insulin receptor substrate/AKT-dependent insulin signaling cascades in insulin-sensitive tissues^7^.

Ceramide is a sphingolipid metabolite and a major component of the plasma membrane and lipoproteins. *De novo* synthesis of ceramide starts from the condensation of serine and palmitoyl CoA. FAs are converted to ceramide via a series of reactions by serine palmitoyltransferase (SPT), 3-ketosphinganine reductase, ceramide synthase, and dihydroceramide desaturase. This *de novo* sphingolipid biosynthesis is activated by excess cellular palmitate and hyperlipidemic conditions in apoE-deficient livers^8,9^. Although this pathway is likely to be activated in the setting of obesity and diabetes, its mechanism of regulation remains uncertain.

SPT is a rate-limiting step in the *de novo* ceramide biosynthesis pathway and is composed of two main subunits, namely, Sptlc1 and Sptlc2, encoding 53- and 63-kDa proteins, respectively^10^. Deficiency of Sptlc1 or Sptlc2 is lethal in mice, and only heterozygous mice or tissue-specific null mice can survive^11,12^. Despite the presence of a third unit, Sptlc3, its function remains unknown^13^. Each subunit is stabilized by forming a dimer or multimer in the endoplasmic reticulum to produce ceramide^14^.

When cellular ceramide is increased by excess FFAs, protein phosphatase 2A is activated and dephosphorylates AKT, leading to inhibition of insulin action^15^. Phosphorylation of the AKT pleckstrin domain prevents binding to 3-phosphoinositides that activate AKT^16^. Taken together, cellular glucose uptake is reduced by ceramide-mediated inhibition of insulin signaling. In this manner, the formation of ceramide is associated with the development of metabolic dysfunctions, such as insulin resistance. Pharmacological or genetic interventions to inhibit the biosynthesis of ceramide will help to alleviate various metabolic abnormalities, including atherosclerosis, cardiomyopathy, and diabetes^17–19^.

The endoplasmic reticulum (ER) is the key organelle responsible for the folding and modification of proteins and protein transport to other cellular compartments^20,21^. ER stress is induced by inflammation and nutrient overload that disrupts ER homeostasis due to the accumulation of unfolded or misfolded proteins in the ER lumen^21,22^. To manage this organelle dysfunction, cells activate a collective signaling cascade referred to as the unfolded protein response (UPR) to relieve ER-associated stress^23^. The conditions that elicit ER stress include glucose or nutrient deprivation, viral infections, disturbance of calcium homeostasis, and excess lipid accumulation^24^. Obesity induces ER stress and plays an important role in the development of insulin resistance and diabetes by activating c-Jun N-terminal kinase (JNK) via activation of inositol-requiring enzyme 1 alpha (IRE1α) and the X-box binding pathway and inhibition of insulin receptor substrate^25,26^.

In this study, we investigated whether ER stress could induce the *de novo* biosynthesis of ceramide and inhibit the insulin response. Sptlc2, a subunit of SPT, was induced by ER stress, and the in vivo effect of ceramide was investigated using liver-specific Sptlc2 transgenic mice. Collectively, the “sphingolipid rheostat,” the balance of ceramide and sphingosine 1-phosphate (S1P) that is necessary for cell survival, is independently regulated by the UPR pathway and contributes to the regulation of metabolic function.

## Materials and methods

### Materials

Tunicamycin, thapsigargin, and dimethyl sulfoxide (DMSO) were obtained from Sigma–Aldrich (St. Louis, MO, USA). Ceramides (C14:0, C16:0, C18:0, C18:1, C20:0, C24:0, and C24:1 ceramides), dihydroceramides (C16:0, C18:0, C24:0, and C24:1), sphinganine, sphingosine, sphingomyelins (SM 16:0, C18:0, and C18:1) and diacylglycerols (C16:0-C16:0, C18:1-C18:1, C16:0-C18:1, C18:0-C18:2, and C18:0-C20:4) were obtained from Avanti Polar Lipids (Alabaster, AL, USA). Insulin was obtained from Eli Lilly Corporate Center (Indianapolis, Indiana, USA). Activating transcription factor 4 (ATF4) and sXBP1 adenoviruses were obtained from Dr. Seung-Hoi Koo (Korea University, Seoul, Korea).

### Primary hepatocyte culture

Primary hepatocytes were isolated from 6–8-week-old C57BL/6 mice (Charles River Laboratories, Wilmington, MA, USA) as described previously^27^. Briefly, after the abdomen was opened, the intestines were displaced to the left, and the portal vein was exposed. The chest was opened, and the hepatic vein was cannulated in a retrograde fashion via an incision in the right atrium with a 24-gauge needle connected to a variable-speed pump that delivered perfusion solution 1 (142 mM NaCl, 6.7 mM KCl, and 10 mM HEPES) at a rate of 8 mL/min. The hepatic portal vein was cut to allow the perfusion fluid to escape. After 2 min, perfusion solution 1 was switched to perfusion solution 2 (66.7 mM NaCl, 6.7 mM KCl, 100 mM HEPES, and 4.8 mM CaCl_2_·2H_2_O) containing 25 mg of collagenase type 4 (Sigma) and 2% bovine serum albumin (BSA) in 50 mL, and the perfusion was continued for another 2–3 min at the same rate. Perfused liver was then excised and transferred to a 60-mm culture dish containing the primary cell culture medium. The liver was broken apart gently with forceps. Cells were counted and plated in medium 199 (Sigma) supplemented with 10% fetal bovine serum (FBS), 1 U/mL penicillin, 1 µg/mL streptomycin, 10 nM dexamethasone, and 23 mM HEPES. The cells were incubated at 37 °C in a 5% carbon dioxide (CO_2_) atmosphere.

### Cell culture

Human hepatocellular carcinoma HepG2 cells were obtained from the American Type Culture Collection (ATCC) (Manassas, VA, USA) and cultured in Dulbecco’s modified Eagle’s medium (DMEM; Welgene, Korea) supplemented with 10% heat-inactivated FBS, 1 U/mL penicillin, and 1 μg/mL streptomycin. The cells were incubated at 37 °C in a 5% CO_2_ atmosphere. Human embryonic kidney cells (HEK-293 cells) were used to generate the recombinant adenovirus and to determine the virus titers. HEK-293 cells were cultured in DMEM supplemented with 10% FBS, 1 U/mL penicillin, and 1 μg/mL streptomycin.

### Generation of liver-specific Sptlc2 transgenic (lSptlc2-Tg) mice and animal experiments

A 1.7-kb fragment of human Sptlc2 cDNA was inserted into the MluI and XhoI sites of the pLIV-7 plasmid. A linearized fragment of the construct containing the promoter, first exon, first intron, and part of the second exon of the human *apoE* gene, the human Sptlc2 cDNA, the adenylation sequence and the hepatic control region of the *apoE/C-I* gene was isolated and microinjected into C57BL/6 J mouse embryos. These embryos were implanted into pseudopregnant C57BL/6J foster mothers. Primers used in the generation of Sptlc2 cDNA with restriction sites were as follows: 5′-AAAAACGCGTATGCGGCCGGAGCCCGGA-3′ and 5′-AAAACTCGAGTCAGTCTTCTGTTTCTTC-3′. Founder animals confirmed by Sptlc2 genotyping were propagated by breeding with C57BL/6J wild-type (WT) mice. Male C57BL/6J mice (8–10 weeks old; Charles River Laboratories) were fed a normal chow diet (NCD; LabDiet, PICO Lab Rodent Diet 20), a 60% high-fat diet (HFD, D12492, Research Diet Inc., NJ, USA), or a high-fructose diet (60% fructose; Central Lab Animal Inc., Korea). ER stress was induced by intraperitoneal injection of tunicamycin (2.5 μg/g body wt) for the indicated times in WT C57BL/6J mice (Charles River Laboratories). At the indicated times, the mice were killed by CO_2_ inhalation, and the livers were harvested to measure sphingolipids and gene expression. To examine the insulin response, an insulin tolerance test (ITT) and glucose tolerance test (GTT) were performed after 9 weeks of feeding. For the GTT, mice were fasted for 16 h, and glucose (2 g/kg body wt) was injected intraperitoneally. For the ITT, mice were starved for 4 h, and insulin (0.75 U/kg body wt, Eli Lilly, Toronto, Canada) was injected intraperitoneally. All procedures were approved by the Gachon University of Medicine Institutional Animal Care and Use Committee (IACUC-R2019011).

### Quantitative reverse-transcription polymerase chain reaction (PCR)

Total RNA from either mouse primary hepatocytes or HepG2 cells was extracted using an easy-spin (DNA free) Total RNA Extraction Kit (iNtRON Biotechnology, Korea), and the mouse livers were homogenized in TRIzol reagent (Invitrogen, Carlsbad, CA, USA). cDNA was synthesized by the iScript cDNA synthesis Kit (Bio–Rad, CA, USA). Real-time PCR analysis was performed in ABI7300 equipment (Applied Biosystems Inc., Carlsbad, CA, USA) using SYBR Green Master Mix (Toyobo, Japan). The mRNA expression levels were expressed as a ratio normalized to those of β-actin^28^. The primers used in this study are shown in Supplementary Table 1.

### Western blotting analysis

HepG2 cells grown to 70% confluence in 6-well plates were treated with the indicated reagents for 16–24 h in DMEM. After treatment, the cells were stimulated with insulin (100 nM) for 10 min. Then, the cells were lysed in cold lysis buffer (10 mM Tris, pH 7.4, 0.5 mM EDTA, 100 μM Na_2_P_2_O_7_, 0.5 mM NaF, 1 mM Na_3_VO_4_, 2% Triton X-100, protease inhibitor, and phosphatase inhibitors). The immunoprecipitates or 30 μg of total protein was applied to SDS–PAGE gels and transferred onto PVDF membranes. Primary antibodies against phospho-AKT1/PKB (Ser473) and AKT/PKB were purchased from Cell Signaling Technology (Danvers, MA, USA). Antibodies against phospho-insulin receptor β subunit (IRβ) (Tyr1162/1163), XBP1 and ATF4 (CREB-2) were purchased from Santa Cruz Biotechnologies (Dallas, TX, USA); IRβ was purchased from Upstate (Burlington, MA, USA); Sptlc1 was purchased from BD Biosciences (Hampton, MA); and Sptlc2 was purchased from Abcam (Cambridge, UK). After incubation with horseradish peroxidase (HRP)-conjugated goat anti-rabbit IgG or goat anti-mouse IgG and IgM (Millipore, Billerica, MA, USA), the blots were developed with enhanced chemiluminescent substrate (Millipore) and detected by a LAS4000 luminescent image analyzer (Fujifilm, Japan). Antibodies against anti-actin (Millipore) were used to normalize the sample loading.

### SPT activity analysis

HepG2 cells were grown to 80–90% confluency and treated with 1.25 μg/mL tunicamycin or 0.5 mmol/L thapsigargin for 24 h. Harvested cells were homogenized in 100 mM HEPES (pH 8.0), 0.5 mM EDTA (pH 8.0), protease inhibitors, and 10% (w/v) sucrose monolaurate. SPT activity was measured with l-serine and palmitoyl CoA as substrates as described previously by Rutty et al.^29^. Briefly, the SPT reaction was initiated by adding 50 µM palmitoyl CoA, 5 mM l-serine, and 20 µM pyridoxal 5′-phosphate to the cell extracts containing 200 µg of protein. After incubation for 60 min at 37 °C, the reactions were terminated by adding 50 μL of NaBH_4_ (5 mg/mL) and reacted for 5 min at room temperature. Lipid fractions were extracted by the addition of methanol/KOH:CHCl_3_ (4:1, v/v), CHCl_3_, alkaline water, and 2 N NH_4_OH, and the lower organic layer was isolated and dried under N_2_ gas. The residues were redissolved in 150 µL of methanol:ethanol:H_2_O (85:47.5:17.5). Then, the samples were analyzed by LC–MS/MS for sphinganine.

### Sphingolipid analysis via LC–MS/MS

For hepatic lipid analysis, HepG2 cells were seeded at a density of 1 × 10^6^ cells/well and incubated at 37 °C for 24 h. After tunicamycin, thapsigargin, or adenoviral infection, the cells were harvested and lysed in RIPA lysis buffer (Thermo Fisher Scientific, Waltham, MA, USA). Mouse livers were isolated and homogenized in PBS with Tissue Lyser II (QIAGEN, Germantown, MD, USA). Internal standards (C17:0-ceramide) were added to the cell extracts containing 1 mg of protein, and sphingolipids were extracted by chloroform/methanol (2:1, v/v) containing 0.01% butylated hydroxytoluene. Phospholipids were saponified by adding KOH at 37 °C for 2 h. Extracts were neutralized by adding acetic acid, and the organic phase was separated and evaporated under N_2_ gas, reconstituted in 100 µL of 0.1% formic acid in methanol, and then analyzed by LC–MS/MS. Ceramides (C14:0, C16:0, C18:0, C18:1, C20:0, C24:0, and C24:1), dihydroceramides (C16:0, C18:0, C24:0, and C24:1), sphinganine, sphingosine, sphingomyelin (C16:0, C18:0, and C18:1), and DAG were separated by HPLC with a C18 column (XTerra C18; 3.5 µm, 2.1 mm × 50 mm) and ionized in positive electrospray ionization mode as described by Yoo et al.^30^. M+/product ions from corresponding sphingolipid metabolites were monitored for multiple reaction monitoring quantification by an API4000Q-trap bench-top tandem mass spectrometer (Applied Biosystems, Framingham, MA) interfaced with an electrospray ionization source.

### Luciferase reporter assay

HepG2 cells (2 × 10^5^ cells/mL) were cultured in 24-well culture plates to 80% confluency for transfection experiments. Cells were transiently cotransfected with pSptlc2-pGL3-Luc and pcDNA3 vector containing ATF4 or sXBP1, respectively, using Lipidofect-P reagent (Lipidomia, Sungnam, Korea) according to the manufacturer’s instructions. An expression vector for Renilla luciferase, pTK-RL, was used to normalize the transfection efficiency. After incubation for 24 h, the cells were washed with PBS once and lysed in passive lysis buffer, and luciferase and Renilla activities were measured using the Dual-Luciferase Assay System (Promega, Madison, WI, USA) according to the manufacturer’s instructions^28^.

### Chromatin immunoprecipitation (ChIP) assay

HepG2 cells were treated with tunicamycin (1.25 µg/ml) for 2 h. Cells were isolated, and ChIP assays were performed according to the manufacturer’s protocol (Abcam, Cambridge, UK). HepG2 cells (3 × 10^6^) were fixed with 1.1% formaldehyde for 10 min at room temperature and stopped with glycine reagent. Chromatin samples, collected by SDS lysis buffer with protease inhibitor, were sheared with sonication for 7 min on ice. The sheared chromatin samples were immunoprecipitated with anti-sXBP1 antibody (Cell Signaling, Danvers, MA, USA) or IgG as a control overnight with rotation at 4 °C. The chromatin/antibody samples were bound to protein A beads, centrifuged to remove insoluble material, and the chromatin/antibody/protein A beads were collected. The DNA was purified with proteinase K. PCR was carried out on 1 µl of DNA using primers for the sXBP1 promoter. The PCR conditions were 95 °C for 5 min; followed by 40 cycles of 95 °C for 30 s, 61 °C for 30 s and 72 °C for 1 min; and a final extension at 72 °C for 7 min. Gels were run on a 1% agarose gel and analyzed with an SML-01 LED Transilluminator (MAESTROGEN, Taiwan).

### Statistical analysis

Data are generally expressed as the mean ± standard error of the mean (SEM). The difference between the 2 groups was analyzed by Student’s *t-test*. A value of less than 0.05 was considered significant.

## Results

### Activation of SPT by ER stress elevates the levels of sphingolipids in HepG2 cells

**Fig. 1:**
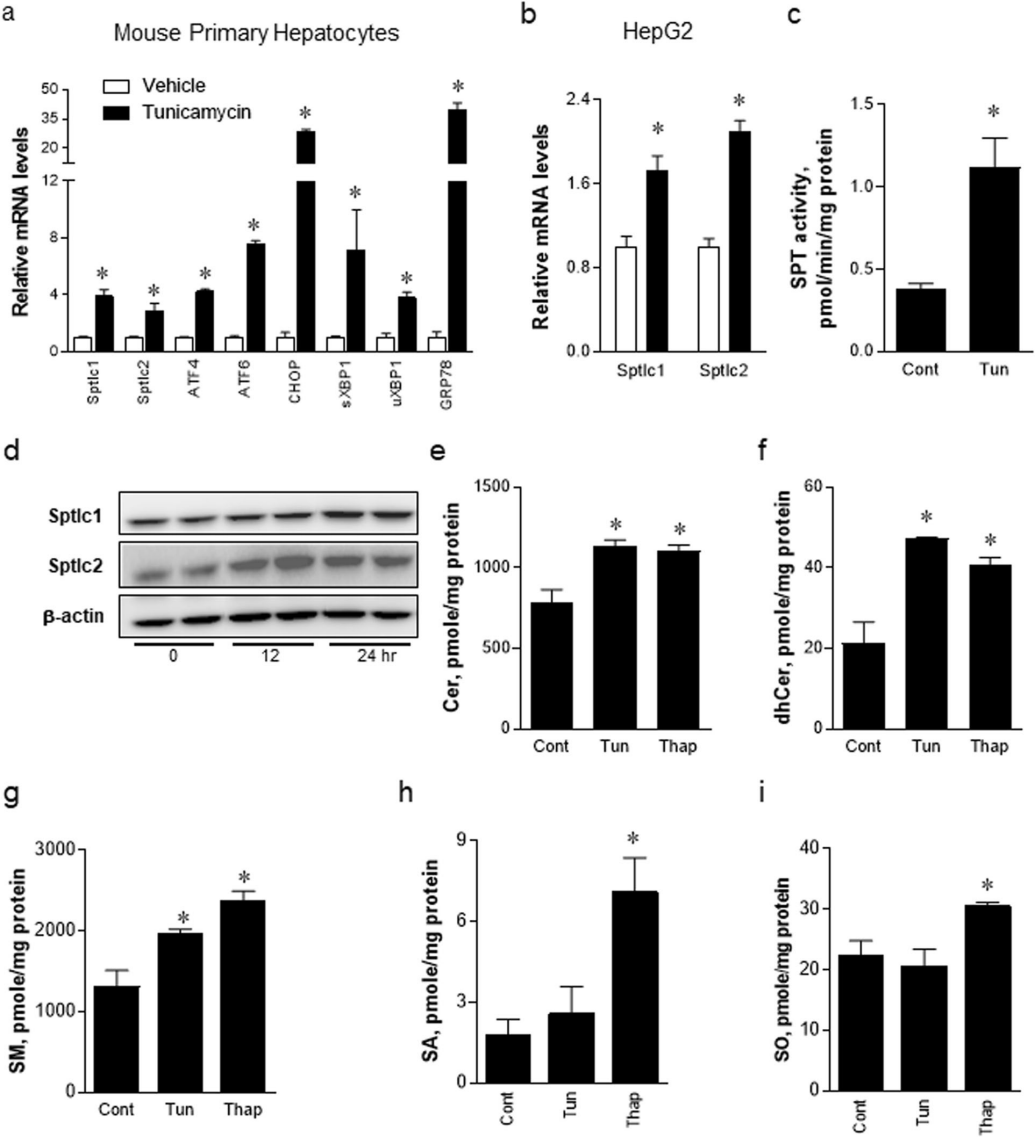
Upregulation of serine palmitoyltransferase long chain base subunit (Sptlc)-1 and Sptlc2 by ER stress inducers in primary hepatocytes and HepG2 cells. Mouse primary hepatocytes or HepG2 cells were treated with dimethyl sulfoxide (DMSO) or tunicamycin (1.25 µg/mL) for 24 h. Isolated mRNA was analyzed by quantitative reverse-transcription polymerase chain reaction (qRT–PCR) (**a**, **b**), and enzyme activity was measured using the cell extracts (**c**). HepG2 cells were treated with tunicamycin for 24 h, and the Sptlc1 and Sptlc2 proteins were analyzed by immunoblotting (**d**). Total ceramides (Cer) (**e**), dhCer (**f**), sphingomyelin (SM) (**g**), sphinganine (SA) (**h**) and sphingosine (SO) (**i**) were analyzed by liquid chromatography-tandem mass spectrometry (LC–MS/MS). *p < 0.05 vs. control. *n* = 3.

To determine whether SPT is activated by ER stress in primary hepatocytes and HepG2 cells, we treated primary mouse hepatocytes and human hepatoma HepG2 cells with tunicamycin, an acute ER stress inducer. Tunicamycin upregulated the expression levels of Sptlc1, Sptlc2 and UPR genes, such as ATF4, CHOP, GRP78, the spliced form of XBP1 (sXBP1) and the unspliced form of XBP1 (uXBP1), in primary mouse hepatocytes. We also observed upregulation of the expression levels of Sptlc1 and Sptlc2 in HepG2 cells (Fig. 1a, b). Furthermore, we measured SPT activity in HepG2 cells treated with tunicamycin and found that SPT activity was increased (Fig. 1c). Next, we examined the expression of Sptlc1 and Sptlc2 in HepG2 cells treated with tunicamycin by immunoblotting (Fig. 1d). The expression of Sptlc1 protein was increased at 24 h, and that of Sptlc2 protein was increased at 12 h. These data suggest that the subunits of SPT, namely, Sptlc1 and Sptlc2, were upregulated by ER stress in hepatocytes.

To assess whether ER stress regulated the metabolism of sphingolipids via the upregulation of SPT, we measured sphingolipid metabolites by LC–MS/MS in HepG2 cells after treatment with tunicamycin or thapsigargin, which are ER stress inducers. Ceramide, dihydroceramide, and sphingomyelin (SM) levels in tunicamycin- or thapsigargin-treated HepG2 cells were significantly elevated (Fig. 1e–g). However, sphinganine (SA) and sphingosine (SO) levels were only elevated in thapsigargin-treated HepG2 cells and were not altered in tunicamycin-treated HepG2 cells (Fig. 1h, i). These results indicated that ER stress upregulates the transcription of SPT subunits, leading to the activation of *de novo* sphingolipid biosynthesis in hepatocytes.

### SPT subunits are upregulated by ER stress in vivo

**Fig. 2:**
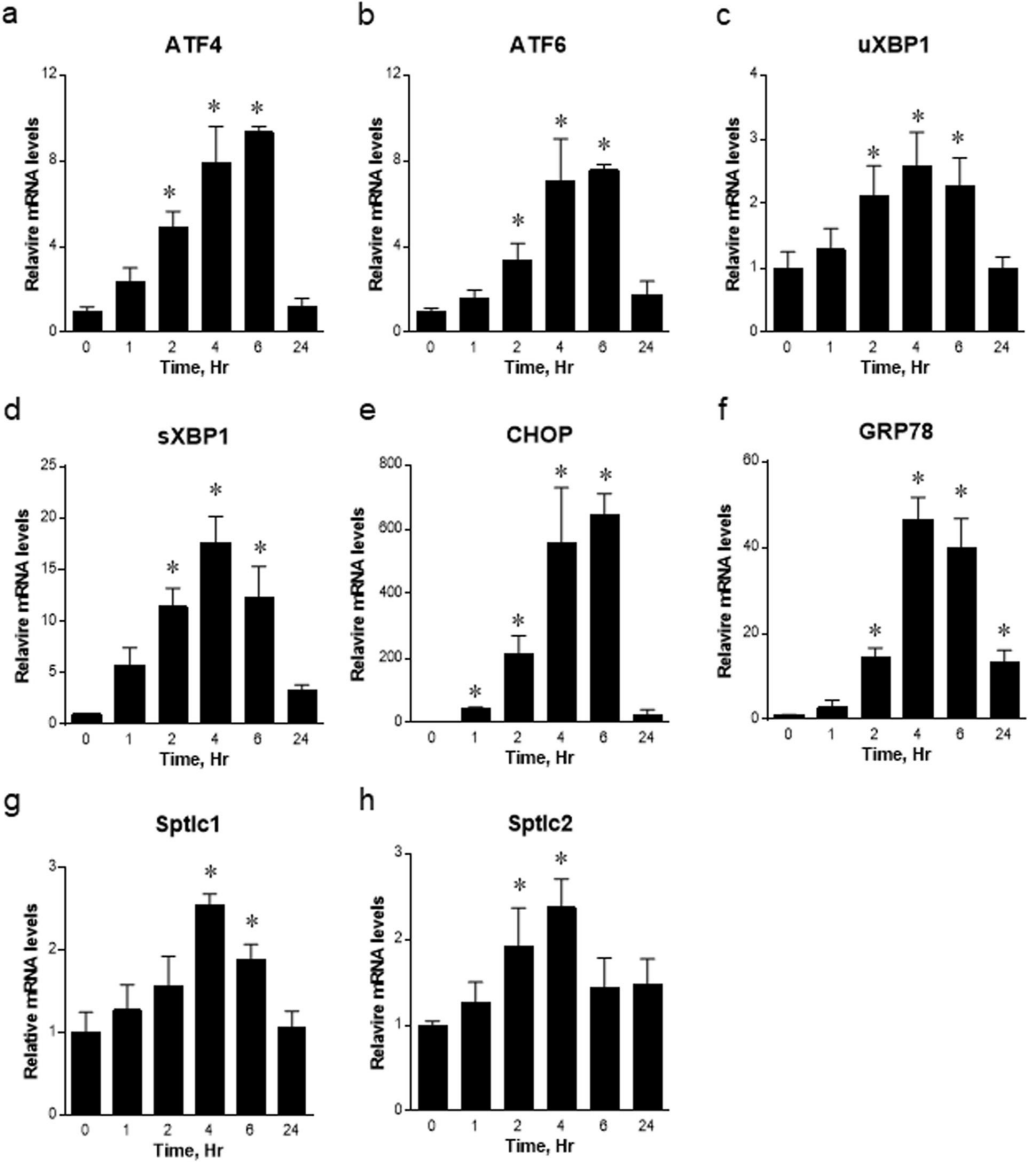
Regulation of the ER stress markers Sptlc1 and Sptlc2 by tunicamycin in wild-type (WT) mice. Tunicamycin (2.5 µg/g body wt) was injected intraperitoneally into C57BL/6 J WT mice. The liver was isolated for mRNA expression analysis at 0, 1, 2, 4, 6 and 24 h after injection. The expression levels of ER stress markers, including ATF4 (**a**), ATF6 (**b**), unspliced XBP1 (uXBP1) (**c**), spliced XBP1 (**d**), CHOP (**e**), GRP78 (**f**), Sptlc1 (**g**), and Sptlc2 (**h**), were measured by qRT–PCR. *p < 0.05 vs. 0 h. *n* = 3.

To determine whether SPT is upregulated by ER stress in vivo, we injected tunicamycin intraperitoneally into C57BL/6 J WT mice, and the livers were isolated at various times postinjection. All ER stress markers, that is, ATF4, ATF6, uXBP1, sXBP1, CHOP, and GRP78, were upregulated by tunicamycin in a time-dependent manner (Fig. 2a–f). In addition, the mRNA expression levels of Sptlc1 and Sptlc2 were upregulated by tunicamycin treatment in the livers of mice treated with tunicamycin in a time-dependent manner (Fig. 2g, h).

**Fig. 3:**
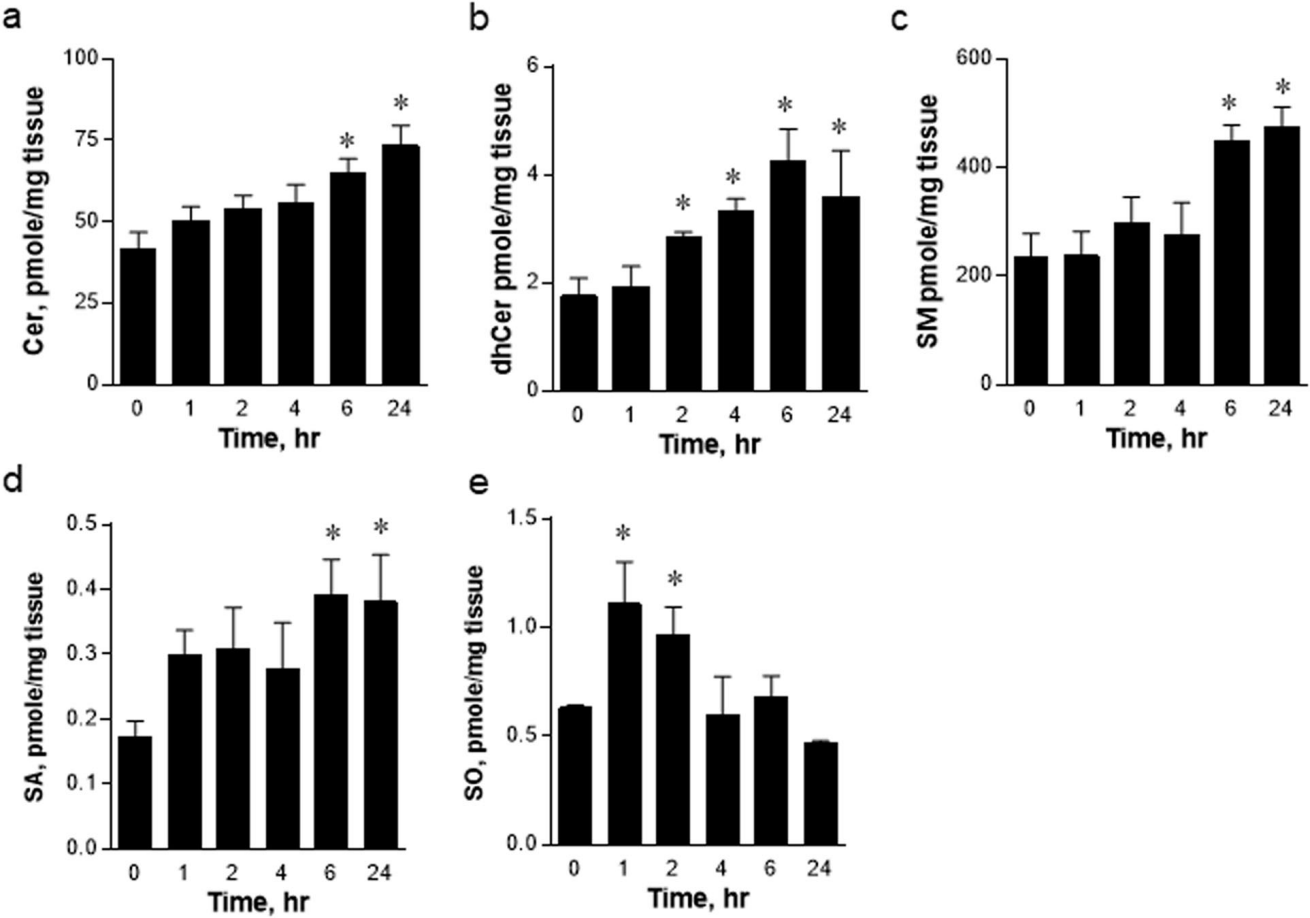
Regulation of sphingolipid synthesis in the liver by tunicamycin in wild-type (WT) mice. Tunicamycin (2.5 µg/g body wt) was injected intraperitoneally into C57BL/6 J WT mice. Ceramides (Cer) (**a**), dhCer (**b**), sphingomyelin (SM) (**c**), sphinganine (SA) (**d**) and sphingosine (SO) (**e**) were analyzed by LC–MS/MS. *p < 0.05 vs. 0 h control. *n* = 3.

To assess whether ER stress regulates sphingolipid metabolism, we measured sphingolipid metabolites in mouse livers treated with tunicamycin by LC–MS/MS. Total ceramide, dihydroceramide, SM, SA, and SO levels in the livers of tunicamycin-treated mice were elevated in a time-dependent manner (Fig. 3a–e). These results suggest that the expression of SPT subunits is upregulated and that *de novo* sphingolipid biosynthesis is activated by ER stress.

### sXBP1 upregulates the expression of Sptlc2 in HepG2 cells

**Fig. 4:**
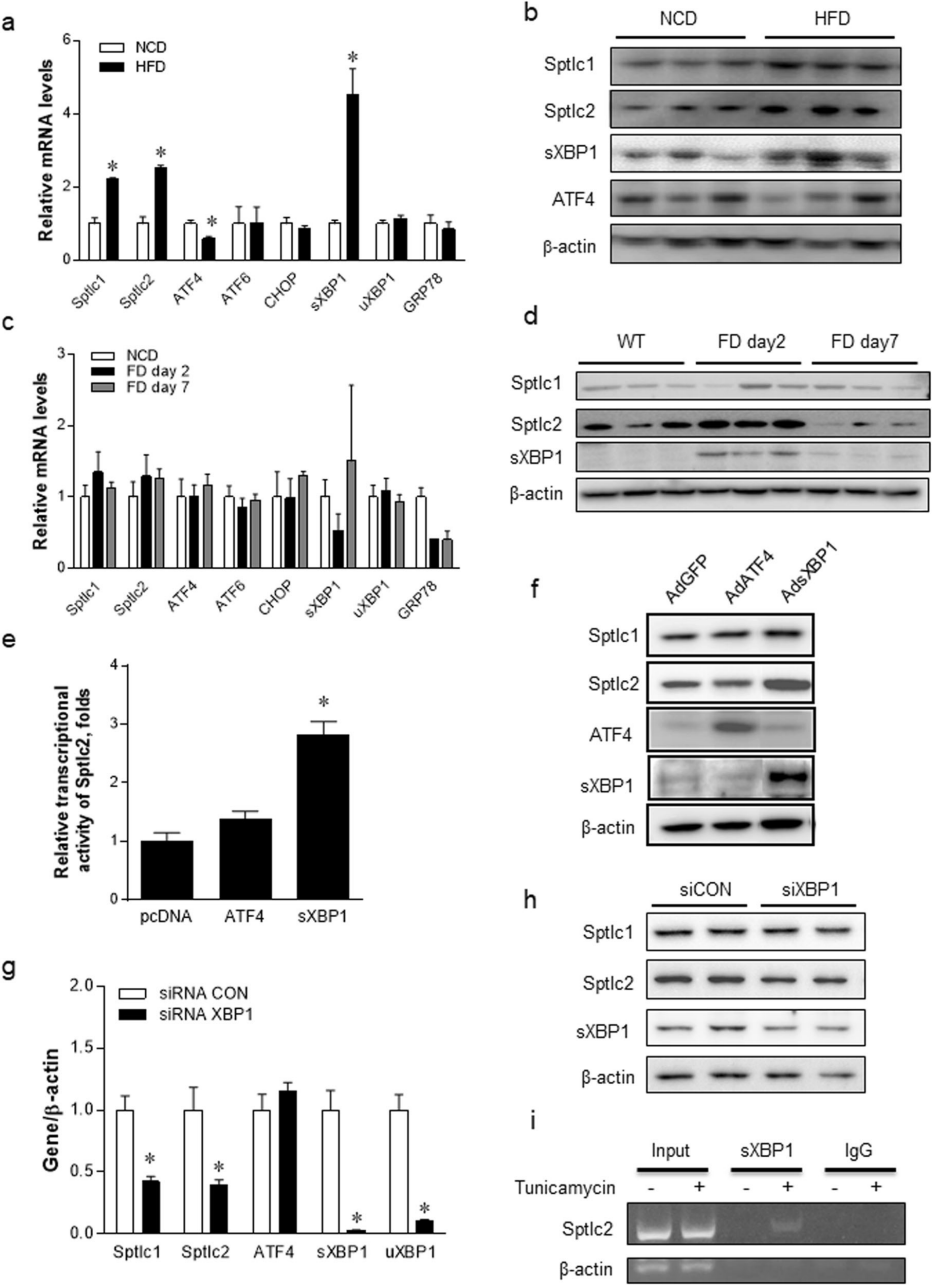
An obesogenic diet upregulates the expression of Sptlc2 via the activation of sXBP1. C57BL/6 J wild-type (WT) mice were fed a normal chow diet (NCD) or a high-fat diet (HFD, 60% of kcal from fat) for 4 weeks, and their livers were collected. Hepatic mRNAs were isolated and analyzed by qRT–PCR (**a**). Protein expression levels of Sptlc1, Sptlc2, sXBP1, and ATF4 were measured by western blotting (**b**). *n* = 5–6, mean ± standard error of the mean (SEM). *p < 0.05 vs. NCD. WT mice were fed a high fructose diet (FD, 60% of kcal from fructose) for 2 and 7 d, and livers were isolated for mRNA (**c**) and protein (**d**) analysis. *n* = 5, mean ± SEM. *p < 0.05 vs. NCD. HepG2 cells were cotransfected with pSptlc2-pGL3-Luc and pcDNA3 containing either ATF4 or sXBP1. Sptlc2 promoter activity was measured by the luciferase assay (**e**). *n* = 5, mean ± SEM. *p < 0.05 vs. pcDNA. HepG2 cells were infected with adenovirus containing ATF4 or sXBP1. Twenty-four hours postinfection, the protein expression levels of Sptlc1 and Sptlc2 were determined by western blotting (**f**). Control and XBP1 siRNA were transfected into mouse primary hepatocytes. After incubating for 48 h, cells were treated with tunicamycin (1.25 µg/mL) for 6 h. mRNA (**g**) and protein (**h**) levels of Sptlc1 and Sptlc2 were analyzed by qRT–PCR and western blotting, respectively. *n* = 3, mean ± SEM. *p < 0.05 vs. siRNA control (CON). HepG2 cells were treated with tunicamycin (1.25 µg/ml) for 2 h. ChIP assays were performed with harvested cells as described in the Materials and Methods and according to the manufacturer’s protocol (**i**).

HFD consumption induces metabolic dysfunction and insulin resistance via the activation of ER stress^31–34^. To examine whether ER stress induced by hyperlipidemic conditions regulates Sptlc2, 8-week-old C57BL/6J WT male mice were fed a normal chow diet (NCD) or a HFD (60% of kcal from fat) for 4 weeks. Mouse livers were isolated, and mRNA expression was measured. Under hyperlipidemic conditions, the expression of Sptlc1 and Sptlc2 was upregulated. HFD feeding increased hepatic sXBP1 activation but decreased ATF4, and no transcriptional changes in other UPR genes were found. Consistent with the mRNA results, the protein expression levels of Sptlc1, Sptlc2, and sXBP1 were increased in the livers from mice fed a HFD compared to levels in the livers from control animals (Fig. 4a, b).

Prolonged feeding of a HFD and a high-sucrose diet are known to increase *de novo* lipid synthesis in the liver via the activation of sXBP1^33,34^. We examined whether XBP1 activation regulates Sptlc2 by giving the mice a fructose diet (FD). WT mice were fed a FD for 2 d or 7 d, and the liver was isolated to measure Sptlc2 expression. While no change was found in the mRNA expression of the SPT subunits and ER stress markers after FD feeding (Fig. 4c), Sptlc2 protein was increased after 2 days of FD feeding (Fig. 4d). As reported previously, the FD induced sXBP1 protein expression after 2 days of FD feeding (Fig. 4d). However, 7-day fructose feeding had no effect on Sptlc1 or Sptlc2. These results indicate that sXBP1 upregulates Sptlc2 under FD-induced physiological ER stress conditions. Next, we examined which UPR pathways are involved in the regulation of Sptlc2 expression and the *de novo* biosynthesis of sphingolipids. To investigate which UPR gene is involved in Sptlc2 upregulation, the promoter of Sptlc2 was cloned into the pGL3-luciferase plasmid to make a reporter construct (pSptlc2-luc) to measure the transcriptional activity of Sptlc2. The pSptlc2-luc reporter plasmid was cotransfected with the pcDNA3.1 plasmid containing ATF4 or sXBP1 into HepG2 cells. Although ATF4 cotransfection had no effect on the expression of Sptlc2, the transcriptional activity of pSptlc2 was enhanced by sXBP1 cotransfection compared to that of the pcDNA3.1 control vector (Fig. 4e). Then, HepG2 cells were infected with adenoviruses (AdGFP, AdATF4, or AdsXBP1) to activate UPR genes. Adenoviral overexpression of sXBP1 increased Sptlc2 protein levels compared with those of ATF4 overexpression or control GFP adenovirus (Fig. 4f). These results suggest that sXBP1 upregulates the expression of Sptlc2. To confirm whether *Sptlc2* is a downstream gene of sXBP1, we suppressed the expression of XBP1 by transfecting XBP1-specific siRNA. Mouse primary hepatocytes were transfected with siRNA control and XBP1-specific siRNA. After 48 h, tunicamycin was administered for 6 h to induce ER stress, and then the cells were harvested to examine mRNA and protein expression levels. We found that XBP1 knockdown suppressed the expression levels of Sptlc1 and Sptlc2 (Fig. 4g, h). We performed a ChIP assay to examine whether Sptlc2 is the direct target of sXBP1. When ER stress was activated by tunicamycin, the Sptlc2 promoter region was immunoprecipitated by sXBP1 antibody (Fig. 4i). In the absence of tunicamycin, no band was detected by sXBP1 antibody-mediated ChIP assay. Therefore, these results suggest that sXBP1 is a direct transcriptional regulator of Sptlc2.

### Liver-specific Sptlc2 transgenic mice fed a HFD exhibit increased insulin resistance

**Fig. 5:**
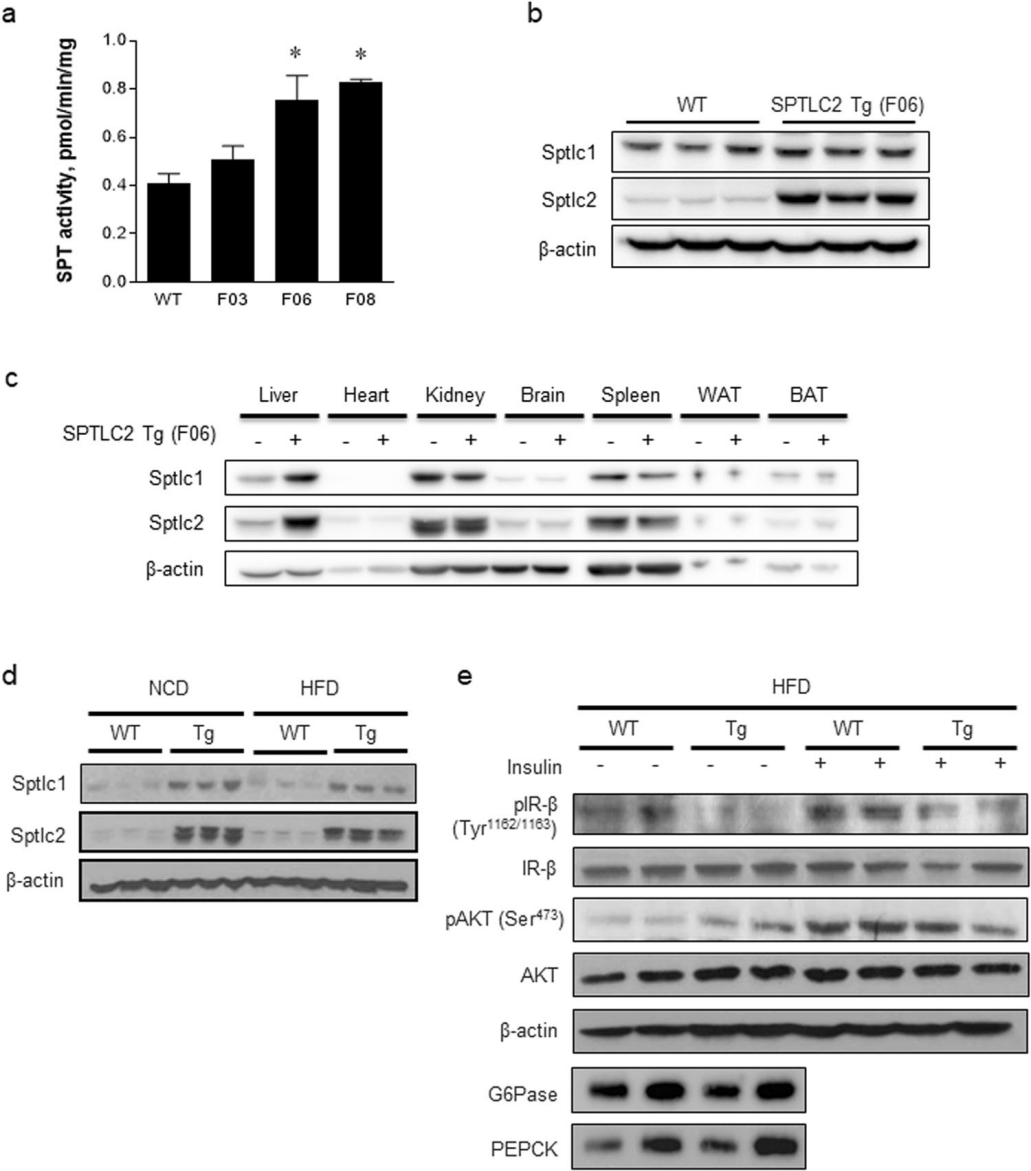
The liver-specific Sptlc2 transgene (lSptlc2-Tg) inhibits insulin signaling. Liver-specific Sptlc2 transgenic mice were constructed, and three germ lines were generated. The hepatic SPT activity of the three germ lines (F03, F06, and F08) was measured (**a**). *n* = 3–4, mean ± SEM. *p < 0.05 vs. wild-type (WT) mice. The protein expression of Sptlc2 in F06 cells was analyzed by western blotting (**b**). The overexpression of hepatic Sptlc2 protein was confirmed (**b**), and there was no overexpression in other organs by immunoblotting (**c**). WT mice and lSptlc2 mice were fed either a normal chow diet (NCD) or a high-fat diet (HFD) for 4 weeks and their livers were isolated to measure the protein expression levels of Sptlc1 and Sptlc2 (**d**). The protein intermediates in the insulin signaling pathway (IR, insulin receptor; AKT) and gluconeogenic proteins (G6Pase, glucose 6-phosphatase; PEPCK, phosphoenolpyruvate carboxykinase) were measured 10 min postinjection of insulin (1 U/kg) by western blotting (**e**).

To investigate the role of Sptlc2 in hepatic glucose/lipid metabolism, we constructed liver-specific Sptlc2 transgenic (lSptlc2-Tg) mice. Three lines of transgenic mice were produced by embryonic injection of pLiv.7 vectors containing human Sptlc2 cDNA (gene ID: BC005123.2). Among them, one line of transgenic mice (F06) was selected for further studies. In the selected line of transgenic mice (lSptlc2-Tg F06), the breeding ability was normal, and the enzyme activity was higher than that in the other lines (lSptlc2-Tg F03 and F08). Hepatic SPT enzyme activity was significantly increased by 2-fold compared with that in the WT (Fig. 5a). Increased Sptlc2 expression was confirmed by immunoblotting analysis (Fig. 5b). We examined whether the Sptlc2 transgene is specific to the liver. We found that the liver showed increased expression of Sptlc2 and that nonhepatic organs showed no change in Sptlc2 expression compared with the expression in WT mice (Fig. 5c).

The correlation between sphingolipid metabolites and insulin response has been reported^35^. Ceramide, a typical sphingolipid metabolite, inhibits serine phosphorylation of the AKT/PKB pathway via the activation of protein phosphatase 2 A (PP2A)^22,23,26^. To test whether hepatic Sptlc2 overexpression decreases the hepatic insulin response, we fed a HFD to mice for 8 weeks. We confirmed Sptlc1 and Sptlc2 expression at the protein level (Fig. 5d). To investigate insulin signaling in the liver, insulin was injected intraperitoneally (50 U/kg body weight) for 10 min before collection of liver tissue. As a result, serine phosphorylation of AKT/PKB was decreased slightly through inhibition of tyrosine phosphorylation of IRβ in HFD-fed lSptlc2-Tg mice compared with that in HFD-fed WT mice (Fig. 5e). In contrast, the expression of gluconeogenic proteins, such as glucose 6-phosphatase (G6Pase) and phosphoenolpyruvate carboxykinase (PEPCK), was not altered. These results suggest that insulin signaling was inhibited by the reduced phosphorylation of IRβ and AKT, but gluconeogenesis was not altered in the lSptlc2-Tg livers.

### Hepatic Sptlc2 overexpression elevates the synthesis of ceramide

We measured selected plasma parameters to determine whether the liver-specific SPTLC2 transgene alters the metabolic state in the plasma. While ALT, AST, total cholesterol, triglycerides, HDL, and LDL were not altered, fasting plasma glucose levels were drastically elevated in lSptlc2-Tg mice (Table 1).

**Table 1:** Plasma parameters in wild-type (WT) and liver-specific Sptlc2 transgenic (lSptlc2-Tg) mice.

	NCD		HFD	
	WT	lSptlc2-Tg	WT	lSptlc2-Tg
Body wt. (g)	31.3 ± 1.0	28.8 ± 1.5	44.4 ± 2.0	46.0 ± 1.7
Glucose (mg/dL)	114.8 ± 11.8	109.2 ± 6.8	163.4 ± 28.7	264.2 ± 39.1*
ALT (U/L)	59.6 ± 6.2	75.2 ± 16.3	119.2 ± 32.7	315.4 ± 81.9
AST (U/L)	105.2 ± 10.4	139.8 ± 14.7	173.5 ± 13.2	229.8 ± 51.1
T-CHO (mg/dL)	92.1 ± 4.0	84.9 ± 3.6	221.3 ± 17.7	224.6 ± 16.4
TG (mg/dL)	107.9 ± 8.0	101.8 ± 6.8	123.2 ± 8.6	110.9 ± 7.7
HDL-C (mg/dl)	60.8 ± 2.8	56.5 ± 2.8	131.7 ± 8.9	128.5 ± 7.3
LDL-C (mg/dL)	8.9 ± 0.8	7.7 ± 0.2	32.1 ± 3.7	35.8 ± 4.9

**Fig. 6:**
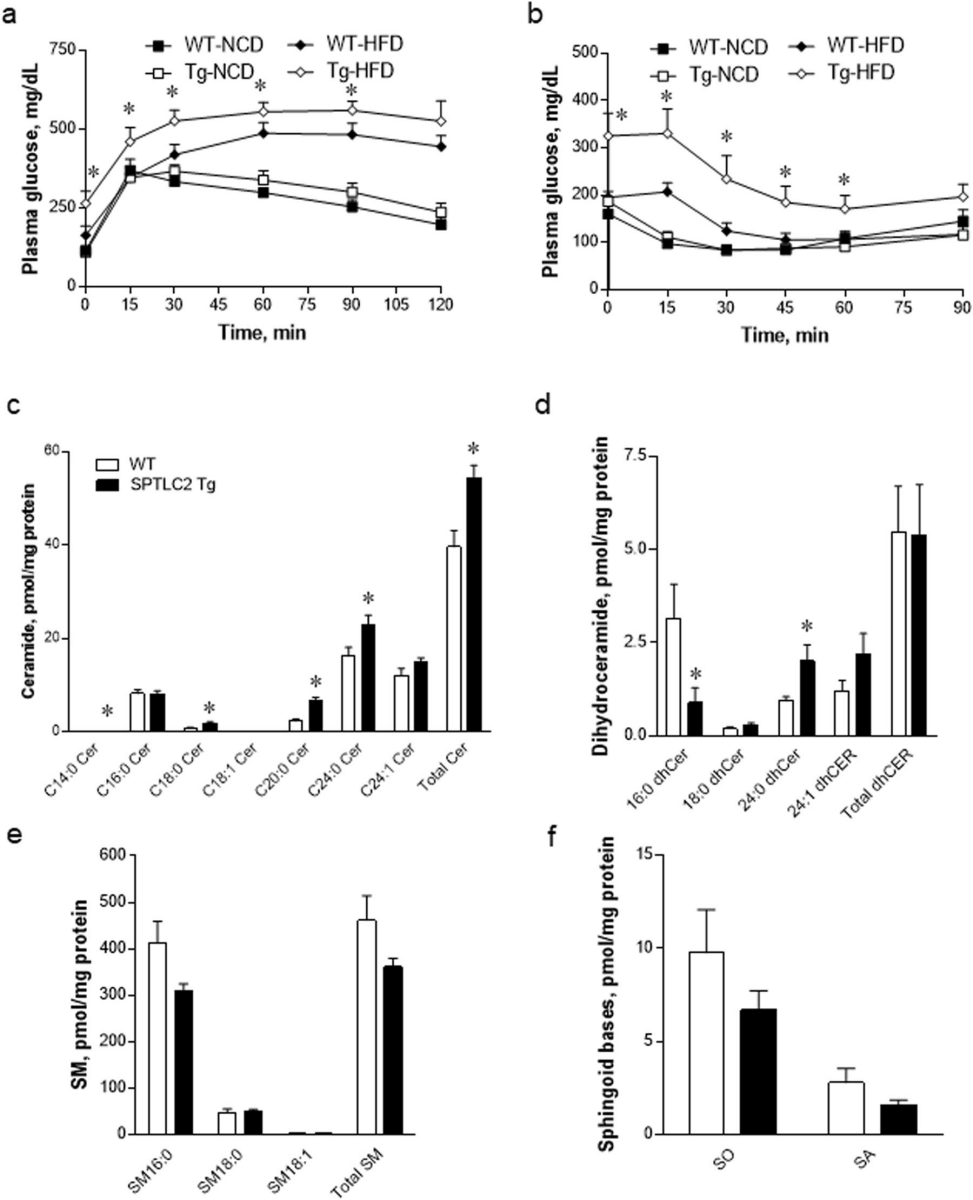
The hepatic Sptlc2 transgene elevates the level of ceramide and induces hyperglycemia. Eight-week-old wild-type (WT) and lSptlc2-Tg mice were fed a normal chow diet (NCD) or a high-fat diet (HFD, 60% kcal fat) for 4 weeks. After 16 h of fasting, glucose (2 g/kg) was injected intraperitoneally into the mice. Blood glucose levels were measured at various times postinjection (**a**). *n* = 5, mean ± SEM. *p < 0.05 vs. WT-HFD. After 4 h of fasting, insulin (0.75 U/kg) was injected intraperitoneally into the mice. Blood glucose levels were measured at various times postinjection (**b**). *n* = 5, mean ± SEM. *p < 0.05 vs. WT-HFD. The levels of ceramide (**c**), dihydroceramide (**d**), SM (**e**), and SO and SA (**f**) were analyzed by LC–MS/MS in the livers from WT and lSptlc2-Tg mice fed a HFD for 4 weeks. *n* = 5, mean ± SEM. *p < 0.05 vs. WT.

To further examine the effects of lSpltlc2-Tg on insulin sensitivity and glucose metabolism, glucose tolerance and insulin tolerance tests were performed. The responses to glucose and insulin were not altered in NCD-fed WT and lSptlc2-Tg mice. In contrast, the glucose intolerance and insulin responsiveness were aggravated in lSptlc2-Tg mice fed a HFD compared with those in WT mice fed a HFD (Fig. 6a, b). These results suggest that lSptlc2-Tg mice fed a HFD have attenuated insulin responses via inhibition of the serine phosphorylation of AKT.

To examine whether hepatic Sptlc2 overexpression affects the synthesis of sphingolipid metabolites, we measured sphingolipid metabolites, including ceramide, dihydroceramide, SM, SO, and SA, in the liver tissues from WT and lSptlc2-Tg mice by LC–MS/MS. We found that total ceramide was increased significantly, but dihydroceramide, SM, SO, and SA were not altered compared with levels in the livers of WT mice (Fig. 6c–f). These findings suggest that hepatic Sptlc2 overexpression elevates ceramide levels in the liver and contributes to the dysregulation of glucose metabolism in the liver.

## Discussion

The endoplasmic reticulum (ER) is an organelle that is responsible for protein folding, maturation, and trafficking. ER stress occurs due to the accumulation of unfolded proteins, which further leads to insulin resistance and metabolic dysfunction^25^. Overnutrition and infection-mediated inflammatory responses are physiological conditions causing ER stress that triggers the UPR to restore ER homeostasis. Under continuous ER stress, lipid homeostasis is perturbed, and cellular messengers, such as DAG and ceramide, lead to the development of insulin resistance in the liver. We found the following: (1) SPT-mediated *de novo* ceramide biosynthesis was activated by ER stress via the XBP1 pathway, (2) ER stress-mediated upregulation of Sptlc2 was responsible for increased ceramide in the liver, and (3) increased ceramide inhibited insulin signaling by decreasing the phosphorylation of IRβ and AKT.

The ER is the major site of protein synthesis, as well as the location for the proper maturation and transportation of correctly folded proteins, along with the Golgi apparatus^25^. In addition, the ER is an essential apparatus in the coordination of metabolism by controlling the synthetic and catabolic pathways of various nutrients. Monitoring of ER status and signaling through the UPR are mediated by three ER membrane-associated proteins, protein kinase RNA-like ER-associated kinase (PERK), IRE1α, and ATF6. In the normal state of the ER, these three membrane proteins bind to BiP/GRP78 and inactivate these signaling proteins^36,37^. The accumulation of improperly folded proteins in the ER results in the release of BiP/GRP78 from these UPR sensors and the activation of PERK and IRE1α. PERK is responsible for the phosphorylation and inactivation of eIF2α, while it activates ATF4, which induces the translation of certain UPR gene clusters^38^. IRE1α is an endoribonuclease that cleaves and generates sXBP1, producing a basic leucine zipper transcriptional activator for the induction of UPR-response genes^39,40^. The UPR also activates ATF6 by promoting its proteolytic cleavage and release from the ER lumen. Liberated ATF6 moves to the nucleus to stimulate a subset of genes, facilitating clearance of misfolded proteins from the ER^41,42^. Improper ER function and activation of the UPR disrupt lipid homeostasis and activate LIPIN2 expression in chronic and acute ER stress conditions via ATF4 activation^32^. DAG, a product of LIPIN2, activates PKCε and inhibits insulin signaling via the inactivation of insulin receptors^35^.

Sphingolipids are basic cellular structural and signaling molecules. Among them, elevation of ceramide by obesity is widely believed to have deleterious effects^16,43–45^ and is associated with toxicity in several tissues, including the liver^46^. *De novo* ceramide biosynthesis has been known to cause the inhibition of insulin signaling. Pharmacological and genetic inhibition of *de novo* ceramide biosynthesis improved glucose intolerance in diet-induced obese (DIO) or heterozygous Sptlc2-deficient mice^47,48^. This finding implied the independent involvement of ceramide in obesity-induced hepatic dysfunction via Sptlc2, a catalytic subunit of SPT. In the liver, sXBP1 regulates the transcription of genes involved in fatty acid synthesis, including stearoyl CoA desaturase-1 (SCD1), acetyl-CoA carboxylase 2 (ACC2), and diacylglycerol acyltransferase 2 (DGAT2), for the accumulation of TG^33^. HFD feeding for 4 weeks caused upregulation of only sXBP1 and the accumulation of lipid droplets in hepatocytes^31^. Early adaptation to ER stress might be initiated by the upregulation of *de novo* ceramide biosynthesis. Induction of acute ER stress by tunicamycin upregulated Sptlc1 and Sptlc2, the subunits of SPT, leading to increased ceramide production. HFD feeding upregulated both SPTLC1 and SPTLC2, while the fructose diet only elevated SPTLC2 (Fig. 4b, d). Even in lSPTLC2-Tg mice, SPTLC1 was upregulated (Fig. 5b, d). The stoichiometry of the SPTLC1 and SPTLC2 subunits is not known exactly, but imbalanced expression of the subunits may be sensed, and the binding partner SPTLC1 seems to be upregulated to restore proper stoichiometry and enzyme activity. These results suggest that the expression of the respective SPTLC subunits is regulated by different insults and that the stoichiometry of the SPTLC1 and SPTLC2 subunits should be maintained to ensure proper enzyme activity response.

Currently, it is not clear whether this ER stress-mediated SPTLC2 induction process is a defensive mechanism to relieve chronic ER stress or a contributing factor for insulin resistance by excess lipids. HFD feeding for 4 weeks increased hepatic Sptlc1 and Sptlc2 in mice. Consistently, short-term feeding with a fructose diet induced Sptlc2 protein expression. A carbohydrate-rich fructose diet activates XBP1 to induce hepatic lipogenesis^33^. We found that sXBP1 upregulated Sptlc2 but that ATF4 did not. Accordingly, only sXBP1 is associated with the regulation of SPT activity and increased ceramide production in hepatocytes. According to a previous report that ATF4 upregulates sphingosine kinase 2^31^, “the sphingolipid rheostat,” which is the balance between ceramide and sphingosine 1-phosphate (S1P), is regulated differently by distinct UPR pathways. The physiological relevance of the synthesis of ceramide or S1P via UPR pathways needs further elucidation.

Overexpression of Sptlc2 had different results in vitro and in vivo. During ER stress, JNK and JNK-mediated serine phosphorylation of IRS-1 via the IRE1α-sXBP1 pathway lead to insulin resistance^49^. In vitro, increased ceramide by adenoviral overexpression of Sptlc2 activated JNK, which phosphorylated IRS-1 at Ser^307^^50^. While insulin signaling was inhibited by Ser phosphorylation of IRS1 in vitro, phosphorylation of IRβ was reduced in the livers of lSptlc2-Tg mice. This result indicated that inhibition of insulin signaling by ER stress is due to ceramide production and decreased AKT phosphorylation. The increase in the levels of ceramide may have decreased the phosphorylation of AKT via protein phosphatase 2A (PP2A)^43^. When adenovirus bearing Sptlc2 was injected into mice fed a NCD, lipid droplets were reduced, and insulin sensitivity and glucose intolerance were improved^50^. This conflicting result is due to the diet (NCD) and nonphysiologically high expression of Sptlc2 in the liver. Hepatic Sptlc2 expression activated VLDL secretion and reduced lipid droplets in the liver. Indeed, the fasting glucose levels were even lower than physiological levels in mice injected with Sptlc2 adenovirus. Under normal conditions, elevated ceramide causes stress in the liver and reduces lipid droplets by increasing VLDL secretion. These results suggest that hepatic glucose/FA metabolism is regulated differently depending on diet condition and the degree of Sptlc2 overexpression.

We observed hyperglycemia in lSptlc2-Tg mice fed a HFD. Decreased hepatic ceramide levels by whole animal or tissue-specific ablation of dihydroceramide desaturase 1 in the liver or adipose tissue ameliorates hepatic steatosis and insulin resistance caused by obesogenic diets^51^. The major cause for the hyperglycemic conditions could be elevated hepatic ceramide levels, but steatosis was not found in lSptlc2-Tg livers (data not shown) since we did not find any changes in the expression of gluconeogenic genes. Interestingly, hyperglycemia was found only in lSptlc2-Tg mice fed a HFD, while no change was found in fasting glucose levels in mice fed a NCD. As reported previously, HFD feeding increases hepatic ceramide levels and causes elevation of fasting glucose levels via the inhibition of insulin signaling^52,53^. In our genetic study, hepatic FA uptake under a HFD supplied substrates for ceramide synthesis and aggravated the dysfunction of glucose metabolism in lSptlc2-Tg livers. We found that only ceramide was elevated in the liver and contributed to the development of hyperglycemia and insulin resistance.

In conclusion, we demonstrated that ER stress-mediated activation of Sptlc2 was regulated by sXBP1. The activation of Sptlc2 increased ceramide levels, inhibiting insulin signaling both in vitro and in vivo. In addition, elevated ceramide decreased pAKT and inhibited the insulin response. The reason for this kind of regulation via sphingolipids might be mainly to reduce the stress on the liver. Taken together, these results indicated that ER stress upregulates Sptlc2 and increases the levels of ceramide, partly contributing to the development of hepatic insulin resistance.

## Supplementary information

Supplementary
