# Supplementary material for: Upregulation of the serine palmitoyltransferase subunit SPTLC2 by endoplasmic reticulum stress inhibits the hepatic insulin response
Source: Exp Mol Med. 2022 May 5;54(5):573–84. doi: 10.1038/s12276-022-00766-4 (PMC9166747; doi:10.1038/s12276-022-00766-4)
Supplement: Supplementary file 1 — Supplementary [file 12276_2022_766_MOESM1_ESM.pdf]

Supplementary Table 1. Primer sequences used in this study.

Gene	Direction	Primer sequences
Mouse β-actin	Forward	5'-GAC GTT GAC ATC CGT AAA G-3'
	Reverse	5'-CAG TAA CAG TCC GCC T-3'
Mouse Sptlc1	Forward	5'-AGT GGT GGG AGA GTC CCT TT-3'
	Reverse	5'-CAG TGA CCA CAA CCC TGA TG-3'
Mouse Sptlc2	Forward	5'-GGA TAC ATC GGA GGC AAG AA-3'
	Reverse	5'-ACC TGG TGT TCT CAG CCA AC-3'
Human Sptlc1	Forward	5'-GGG TGG CAG GTC TTT TGT AAT T-3'
	Reverse	5'-CAA CAC TGC AAA AAT ACC TGG -3'
Human Sptlc2	Forward	5'-GAG ACG CCT GAA AGA GAT GG-3'
	Reverse	5'-TGG TAT GAG CTG CTG ACA GG-3'
Mouse ATF4	Forward	5'-TGG AAA CCA TGC CAG ATG AG-3'
	Reverse	5'-GAT GGC CAA TTG GGT TCA CT-3'
Mouse ATF6	Forward	5'-TCG CCT TTT AGT CCG GTT CTT-3'
	Reverse	5'-GGC TCC ATA GGT CTG ACT CC-3'
Mouse GRP78	Forward	5'-TGC AGC AGG ACA TCA AGT TC-3'
	Reverse	5'-TTT CTT CTG GGG CAA ATG TC-3'
Mouse CHOP	Forward	5'-CAG TCA TGG CAG CTG AGT CC-3'
	Reverse	5'-TAG GTG CCC CCA ATT TCA TC-3'
Mouse uXBP-1	Forward	5'-TAT CCT TTT GGG CAT TCT GG-3'
	Reverse	5'-AAA GGG AGG CTG GTA AGG AA-3'
Mouse sXBP-1	Forward	5'-CTG AGT CCG AAT CAG GTG CAG-3'
	Reverse	5'-GGG AGT GGA GTA AGG CTG GT-3'
Mouse PEPCK	Forward	5'-CCC CTT GTC TAT GAA GCC CT-3'
	Reverse	5'-GCC CTT GTG TTC TGC AG-3'
Mouse G6Pase	Forward	5'-TCT GTC CCG GAT CTA CCT TG-3'
	Reverse	5'-GTA GAA TCC AAG CGC GAA AC-3'
